# Prevalence of depression, anxiety and associated factors among patients with dental disease attending outpatient department in Addis Ababa public hospitals, Addis Ababa, Ethiopia: a multicenter cross-sectional study

**DOI:** 10.1186/s12903-021-02012-1

**Published:** 2021-12-09

**Authors:** Bekele Seifu, Niguse Yigzaw, Kibrom Haile, Zahira Reshid, Henock Asfaw

**Affiliations:** 1 Eka Kotebe General Hospital, Ethiopian Treatment Center of COVID-19, Addis Ababa, Ethiopia; 2 School of Medicine, College of Health and Medical Science, University of Gonder, Gonder, Ethiopia; 3 Amanuel Mental Specialized Hospital, Addis Ababa, Ethiopia; 4 School of Nursing and Midwifery, College of Health and Medical Science, Haramaya University, Haramaya, Ethiopia

**Keywords:** Depression, Anxiety, Dental diseases, Public hospitals, Addis Ababa, Ethiopia

## Abstract

**Background:**

Anxiety and depression are widespread mental health problems in many populations. These problems can be major barriers to dental care and may be led to poor oral health.

**Objectives:**

To assess prevalence of depression, anxiety and associated factors among patients with dental disease in Addis Ababa public hospitals outpatient department, Addis Ababa, Ethiopia, 2019.

**Methods:**

An institution based cross sectional study was conducted from May 06 to June 06, 2019 among patients with dental disease attending outpatient department in Addis Ababa city administration public hospitals. Multistage sampling method was used to select study participants. Hospital Anxiety and Depression scale was used to assess anxiety and depression. Face to face interview was used to collect data and the collected data was entered into EPI data version 3.1 and analysis was done using SPSS (Statistical Package Software for Social Sciences) version 20. Bi-variable and multivariable binary logistic regression was carried out. Strength of association was determined using odds ratio with 95% CI (Confidence Interval) and *p* value less than 0.05 was considered as statistically significant association in the final model.

**Results:**

From the total of 845 participants, 833 were studied with response rate of 98.6%. The median age of the respondent was 32 years with interquartile range (26–41 years). The prevalence of anxiety and depression were found to be 33.9% and 29.2% respectively. Being female [AOR (Adjusted Odds Ratio) 2.70 (95% CI 1.86, 3.89)], tooth extraction [AOR 3.24 (95% CI 2.11, 4.97)], history of repeat visit to dental clinic [AOR 3.21 (95% CI 2.25, 4.58)], chronic disease [AOR 2.95 (95% CI 1.98, 4.38)] and current alcohol use [AOR 3.40 (95% CI 2.28, 5.09)] were significantly associated with anxiety among patients with dental disease. Being female [AOR 2.22 (95% CI 1.53, 3.23)], Elementary educational status [AOR 2.15 (95% CI 1.28, 3.58)], periodontitis [AOR 1.74 (95% CI 1.18, 2.72)],history of repeated visit to dental clinic [AOR 4.07 (95% CI 2.84, 5.84)], current use of alcohol [AOR 4.01 (95% CI 2.68, 6.00)], current cigarette use [AOR 3.15 (95% CI 1.42, 7.00] and irregular tooth brushing [AOR 2.22 (95% CI 1.53, 3.23]were significantly associated with depression among patients with dental disease.

**Conclusion:**

Anxiety and depression were high among people with dental disease. Tooth extraction and having chronic disease were significantly associated with anxiety. Elementary educational status, periodontitis, current cigarette smoking and irregular tooth brushing pattern were significant association with depression. History of repeat visit to dental clinic, current alcohol use and female sex were significantly associated with both depression and anxiety. Based on the finding of this study early screening and treating of anxiety and depression, also identifying those associated factors are important at dental clinic.

## Introduction

Oral diseases are serious and the most prevalent chronic diseases globally. The economic impact for its treatment accounts to be 5% of total health care costs. Dental diseases are high among disadvantaged and poor population groups both in developed and developing countries. The common dental diseases are periodontal disease, pulpits, gingivitis, oral mucosal lesion, root stamp and oral manifestation of Human Immune virus (HIV) infection. Among those, periodontal disease is the eleventh most prevalent dental disease worldwide and its major causes are behavioral factors (poor oral hygiene and cigarette smoking) [1–3].

Depression is a mental disorder which characterized by clinical features of low mood, loss of interest in activity, unintended weight change, sleep problem, psychomotor agitation or slowness, decreased energy, feeling worthless and hopeless, having difficulty of decision making and having repeated thoughts of harming oneself or suicide [4].

Anxiety is an alerting signal; it warns of impending danger and enables a person to take measures to deal with a threat and can be viewed as a family of related but distinct mental disorders, which include panic disorder, agoraphobia, specific phobia, social anxiety disorder or phobia, and generalized anxiety disorder [5].

The World Health Organization (WHO) report stated that, the prevalence of anxiety disorder in the global population in 2015 is estimated to be 3.6%, and it is more common among females 4.6% than males 2.6% at the global level. Generally, 264 million people are living with anxiety disorders globally and its prevalence rate is substantially high in general population [6].

Depression and anxiety are common mental disorders which have adverse effect on person’s quality of life. People with both clinically significant depression and anxiety often have greater severity of diseases in general, less control of respiratory problem(asthmatic), longer bed occupancy, more visit primary health care, and more consume medications(pain killer) and they are also associated with stress and/or anxiety, so they may change course and prognosis of the other medical illnesses [7, 8].

Study showed that, 73%-79% of individuals have experienced at least some anxiety during dental treatment, whereas it has been estimated that one in every five patients who attend for checkups to dentist or physician experience clinically significant symptoms of depression [9, 10].

Dental health professionals including dental surgeons spend a lot of time treating patients who present with depression and anxiety or with physical illnesses which show remarkable underlying emotional problems [11]. However, some recognizable psychopathologies like depression and anxiety are frequent in patients visiting to dental outpatient department, more of which go unidentified, unconcerned and hence untreated [12].

A variety of studies done across the world revealed that the prevalence of depression and anxiety among patients with dental disease were Sweden [13], 4.8% and 6.8%, Pakistan, [14] 11.2% and 22.5%, Turkey [15] 41% and 36% and Finland [16] 26% and 10% respectively.

The most frequently reported factors correlated with depression and anxiety among patients with dental diseases were being females [15, 17, 18], older age [18], less education [18], less income [16, 18], not having a dental visit within the year [18], periodontitis [19], poor oral hygiene [20], smoking [19] and alcohol use [21].

There is paucity of epidemiologically reliable data on depression and anxiety among patients with dental diseases in Ethiopia. Therefore current study was aimed to assess the prevalence of depression, anxiety and associated factors among patients with dental disease attending outpatient department in Addis Ababa public hospitals.

## Materials and methods

### Study area and period

Addis Ababa is a capital city of Ethiopia. It is located 9.02 latitude and 38.75 longitudes and it is the largest city with ten sub cities [22]. There are twelve public hospitals in the city. Among this ten have large dental outpatient services in the city which serves five days each week. The study was conducted among four randomly selected hospitals with an average monthly patient flow of, in Zewditu Memorial referral hospital, Yekatit-12 hospital Medical College, Menelik II hospital, and Ras Desta Damtew Hospital. Totally, four hospitals host for an average of 2545 dental outpatients per month. The data was collected from May 06 to June 06, 2019.

### Study design and population

Institutional based quantitative cross sectional study design was employed. All patients with dental disease attending outpatient dental clinics in Addis Ababa public hospitals whose age is 18 years and above and critically ill patients were excluded.

### Sample size determination and sampling procedure

The optimum number of samples required for the study was estimated using single population proportion formula considering the assumption of proportion of the prevalence of anxiety and depression among adult dental out patients was unknown in Ethiopia; P = 50% = 0.5 was used, d = margin of tolerable error tolerated = 5% = 0.05 Z = 95%confidence interval (CI) = 1.96.

Where:

$${\text{n}}_{{\text{i}}} = \frac{{\left( {{\text{Z}}\alpha^{1/2} } \right)^{2} {\text{p}}(1 - {\text{p}})}}{{{\text{d}}^{2} }}$$

$$n{\text{i}} = \frac{{(1.96)^{2} \times (0.5) \times (1 - 0.5)}}{{(0.05)^{2} }} = 384$$

Since this was multistage sampling method multiplying the result by 2 design effect i.e. *n*i = 384 × 2 = 768. Then considering 10% non-response rate, 768 × 10% = 77. Therefore, 768 + 77 = 845 was final sample size.

**Fig. 1:**
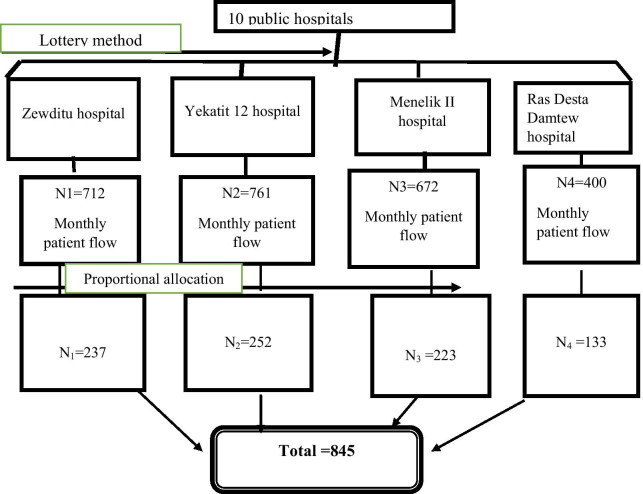
The schematic presentation of the sampling procedure for the study on the prevalence of depression, anxiety and associated factors among patient with dental disease attending outpatient department in Addis Ababa public hospitals, 2019

Concerning sampling procedure, the study was carried out by using multi stage method. Lottery method was used to select four hospitals out of ten public hospitals providing dental care under Addis Ababa city administration. Then systematic random sampling technique was used to select study participants at dental outpatient clinics from these hospitals during the study period. Samples were taken proportionally from each included hospital (Fig. 1).

### Data collection tool and procedure

The data was collected by using standard tools. Socio-demographic variables were collected by using structured questionnaire. Clinical factors were collected by semi-structured questionnaires. For substance-related factors, ASSIST which is a brief screening questionnaire developed by the World Health Organization to find out about people’s use of psychoactive substances will be used to assess current and ever substance use history of the subject. Oral malodor and oral hygiene habit were assessed by structured questions.

The dependent variables such as depression and anxiety were measured by Hospital Anxiety and Depression scale (HADS). HADS is a 14-item questionnaire, commonly used to screen symptoms of anxiety and depression, has 7-item sub-scales for each. It was previously being translated into Amharic and validated in Ethiopia and it has been used for institutional based study. Its internal consistency was 0.78 for anxiety, 0.76 for depression subscale and 0.87 for the full scale of HADS. The intra-class correlation coefficient was 80%, 86%, and 84% for HADS-A, HADS-D subscales, and HADS, respectively. The scales use a cut off score ≥ 8 for both anxiety and depression [23]. Social support was measured by Oslo social support scale(oslo-3), the sum score scale ranging from 3–14, which has three categories: poor support 3–8, moderate support 9–11 and strong support 12–14 [24] and income was measured by 2017 updated world Bank Atlas method [25].

### Data processing and analysis

The collected data was entered into EPI data version 3.1 and analysis was done using SPSS version 20. Bi-variable and multivariable binary logistic regression was carried out. Association was checked between dependent and independent variables.

Independent variables with *p* value less than 0.20 in bi-variable logistic analysis was fitted in to multivariable logistic regression to identify independently associated factors in the model; Strength of association was determined by using odds ratio with 95% CI and *p* value less than 0.05 was considered as statistically significant association in the final model.

### Data quality control

The questionnaire having different sections was translated from English to Amharic language by psychiatry professionals. Then it was translated back to English by language expert to check for consistency and understandability of the tool. One day training was given for data collectors and supervisors on how to use the questionnaire, sampling techniques, ethical principles, and data managements and how to identify participants and referral process. Pretest of data collection tools was carried out on 5% of the sample before conducting the study with Cronbach’s α = 0.70 and 0.71 for anxiety and depression respectively. Supervision was held regularly during data collection period on completeness and consistency of collected data.

### Ethical consideration

Ethical clearance was obtained from the Ethical Review Board of University of Gondar, Amanuel Mental Specialized Hospital and Addis Ababa Public Health Research and Emergency Management Directorate. A formal letter of support was obtained from Amanuel Mental Specialized Hospital and Addis Ababa city Administration health bureau and submitted to Zewditu Memorial hospital, Yekatit-12 hospital Medical College, Menelik II hospital and Ras Desta Damtew hospital. Before questionnaire was administered to any eligible participant written and verbal consent was obtained from each study participant after the study objective explained to them in detail by the data collectors. The right was given to the study participants to refuse or discontinue participation at any time they want and the chance to ask any thing about the study. For the purpose of confidentiality, the participant’s name and personnel information was maintained at the time of data collection and assured throughout the study period. Data collectors were putted their signature for verbal consent they obtained for the interview from the respondents. For depressed and anxious cases, recommendation was given to link to psychiatric unit of each hospital and those who scored beyond the cut off point to both depression and anxiety were linked to psychiatric service for further evaluation and treatment.

## Results

### Socio-demographic characteristics

A total of 845 study participants were studied, giving a response rate of 98.6%. The median age of the respondent was 32 years with interquartile range (26–41 years). More than half of study participants 466 (55.9%) were females. Four hundred forty-two (53.1%) were married. About 283 (34%) of study participants had completed high school. Regarding occupational status of participants 378 (45.4%) was private business worker and the median monthly income of participant’s was 2000 Ethiopian birr and interquartile range (545–3000 Ethiopian birr) as shown in Table 1.

**Table 1:** Socio demographic variables among people with dental disease in outpatient department at Addis Ababa public hospitals of Addis Ababa, Ethiopia, 2019 (n = 833)

Variables	Category	Frequency	Percentage (%)
Age group	18–29	331	39.7
	30–44	329	39.5
	> 45	173	20.8
Sex	Female	466	55.9
	Male	367	44.1
Marital status	Married	442	53.1
	Single	311	37.3
	Divorced	40	4.8
	Widowed	40	4.8
Educational status	No formal education	121	14.5
	Elementary	242	29.1
	High school	283	34
	College /university	187	22.4
Occupation status	Private business	378	45.4
	Government employee	224	26.9
	Jobless	121	14.5
	House wife	58	7
	Student	52	6.2
Average monthly income	< 1627 Ethiopian Birr	390	46.8
	≥ 1627 Ethiopian Birr	443	53.2

### Clinical and psychosocial characteristics of participants

Regarding clinical factors, more than half of dental disease diagnosis were periodontitis accounts 483 (58%) and tooth extraction accounts 587 (60.9%). Respondents who suffered from dental trauma were 178 (21.4%), and having chronic disease was 178 (21.4%). The majority of study participants who attend dental clinic 609 (67.5%) were new patients. Most of study participants 748 (89.8%) had no dental checkup once or greater than once per year. Respondents those who had irregular tooth brushing pattern and worry about their oral bad odor were 463 (55.6%) and 224 (26.9%) respectively. Participants having family history of mental illness were 67 (8%). Moderate social support of the study participant was 359 (43.1%) as shown in Table 2.

**Table 2:** Clinical and psychosocial factors among people with dental disease in outpatient department at Addis Ababa public hospitals Addis Ababa, Ethiopia, 2019 (n = 833)

Variables	Category	Frequency	Percentages (%)
Type of dental disease	Periodontitis	483	58
	Pulpitis	202	24.2
	Root stamp	81	9.7
	Gingivitis	67	8
Type of procedure/service	Extraction	587	60.9
	Scaling	243	29.2
	Filling	83	10
Family history	No	766	92
	Yes	67	8
Dental trauma	No	655	78.6
	Yes	178	21.4
Chronic disease	No	655	78.6
	Yes	178	21.4
Hx of visiting dental clinic	New	562	67.5
	Repeat	271	32.5
Dental checkup/year	No	748	89.8
	Yes	85	10.2
Tooth brushing habit	Irregular	463	55.6
	Regular	370	44.6
Oral bad breath	No	609	73.1
	Yes	224	26.9
Social support	Poor social support	335	40.2
	Moderate social support	359	43.1
	Strong social support	139	16.7

### Substance use history of participants

**Fig. 2:**
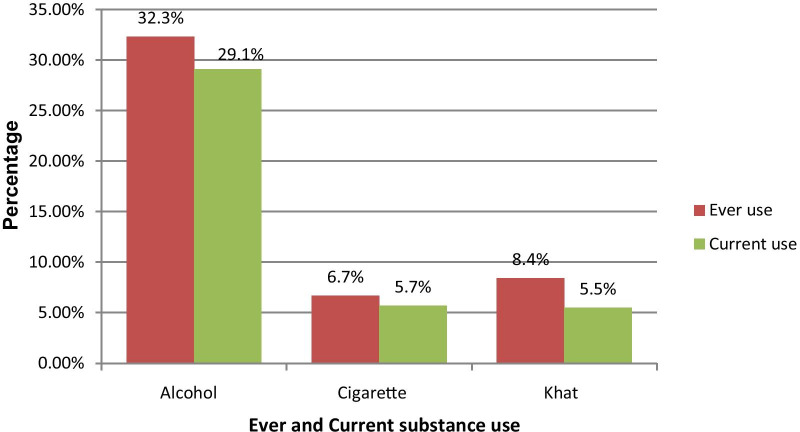
Distribution of substance use among people with dental disease in outpatient department at Addis Ababa Public hospitals Addis Ababa, Ethiopia, 2019 (n = 833)

Among the total study participants 29.1% of them were reported a history of current alcohol use, 5.7% were cigarette smokers and 5.5% were khat chewers (Fig. 2).

### Prevalence of anxiety and depression among patients with dental disease

This study revealed that the prevalence of anxiety and depression were 33.9% (95% CI 30.6–37.2) and 29.2% (95% CI 26.1–32.3) respectively.

### Factors associated with anxiety among people with dental disease

Bi-variable analysis was done for each explanatory variable. Socio-demographic factors such as sex, occupational status and average monthly income fulfilled the minimum requirement. Clinical factors; Dental procedures, history of visiting dental clinic and having chronic disease also fulfilled the minimum criterion. Substance use factors; current and life time alcohol, khat and cigarette use were variables satisfied the minimum requirement (p < 0.2 significance level) for further multivariable logistic analysis in anxiety. Multivariable logistic analysis was computed. It showed that, the model adequately fits the data for anxiety as *p* value from Hosmer and Lemeshow test was 0.772. During multivariable analysis; sex, tooth extraction, chronic disease, history of repeat visit to dental clinic and current Alcohol use were significantly associated with anxiety.

According to the result of this study being female was 2.7times [AOR 2.70 (95% CI 1.89, 3.89)] more likely to develop anxiety as compared to male. The odds of having anxiety disorder among study subjects with tooth extraction was 3.24times [AOR 3.24 (95% CI 2.11, 4.97)] higher when compared individuals with tooth scaling. Those who have history of repeated visit to dental clinic was 3.21times [AOR 3.21 (95% CI 2.25, 4.58)] more likely to have anxiety as compared with new patients visiting dental clinic. Having chronic diseases were 2.95times [AOR 2.95 (95% CI 1.98, 4.38)] higher to have anxiety as compared to those who have no chronic disease. Individuals who have reported current alcohol use was 3.40 times [AOR 3.40 (95% CI 2.28, 5.09)] as compared with non-users as shown in Table 3.

**Table 3:** Bi-variable and multivariable logistic analysis of associated factors with anxiety among people with dental disease in outpatient department at Addis Ababa public hospitals, Addis Ababa, Ethiopia, 2019 (n = 833)

Variables	Category	Anxiety		COR (95% CI)	AOR (95% CI)
		Yes	No		
Sex	Female	190 (22.8%)	276 (33.1%)	2.06 (1.52–2.77)	2.70 (1.86–3.89) *
	Male	92 (11.01%)	275 (33.01%)	1.00	1.00
Type of procedure/service	Extraction	216 (25.9%)	291 (34.9%)	3.66 (2.50–5.34)	3.24 (2.11–4.97) *
	Filling	25 (3.0%)	58 (6.9%)	2.12 (1.20–3.78)	1.52 (0.78–2.95)
	Scaling	41 (4.9%)	202 (24.2%)	1.00	1.00
Occupational status	Private business	117 (14.0%)	261 (31.3%)	1.00	1.00
	Government employee	70 (8.4%)	154 (18.4%)	1.00 (0.71–1.45)	0.91 (0.58–1.40)
	Jobless	44 (5.2%)	77 (9.2%)	1.28 (0.83–1.96)	1.21 (0.69–2.11)
	Student	21 (2.5%)	31 (3.7%)	1.51 (0.83–2.73)	1.34 (0.65–2.76)
	House wife	30 (3.6%)	28 (3.3%)	2.39 (1.37–4.18)	1.04 (0.53–2.05)
Chronic disease	Yes	100 (12.0%)	78 (9.3%)	3.33 (2.37–4.69)	2.95 (1.98–4.38) *
	No	182 (21.8%)	473 (56.6%)	1.00	1.00
History of visiting dental clinic	Repeat	137 (16.4%)	134 (16.0%)	2.94 (2.17–3.99)	3.21 (2.25–4.58) *
	New	145 (17.4%)	417 (50.0%)	1.00	1.00
Current alcohol use	Yes	137 (16.4%)	105 (12.6%)	4.00 (2.93–5.50)	3.40 (2.28–5.09) *
	No	145 (17.4%)	446 (53.5%)	1.00	1.00
Current cigarette use	Yes	20 (2.4%)	17 (2.04%)	2.40 (1.24–4.65)	1.71 (0.74–3.94)
	No	262 (31.4%)	534 (64.1%)	1.00	1.00
Current Khat use	Yes	24 (2.88%)	22 (2.64%)	2.24 (1.23–4.07)	0.93 (0.32–2.69)
	No	258 (30.97%)	529 (63.5%)	1.00	1.00
Life time khat use	Yes	35 (4.2%)	35 (4.2%)	2.09 (1.28–3.42)	1.64 (0.70–3.80)
	No	247 (29.6%)	516 (61.9%)	1.00	1.00
Life time alcohol use	Yes	112 (13.4%)	157 (18.8%)	1.65 (1.22–2.24)	0.94 (0.67–1.54)
	No	170 (20.4%)	394 (47.2%)	1.00	1.00
Average monthly Income	Below poverty line(< 1627 Ethiopian birr)	153 (18.3%)	237 (28.4%)	1.57 (0.18–2.09)	1.21 (0.80–1.82)
	Above poverty line(≥ 1627 Ethiopian birr)	129 (15.4%)	314 (37.6%)	1.00	1.00

^*^Significant association (*p* value < 0.0001), Hosmer and Lemeshow test = 0.772. Chronic disease = hypertension, renal disease, HIV/AIDS & diabetes

### Factors associated with depression among people with dental disease

Bi-variable analysis for depression was made for each explanatory variable. Socio-demographic variables; sex, educational status and average monthly income were set minimum requirement. Clinical variables like dental diagnosis and history of visit to dental clinic were satisfied minimum criteria. Behavioral and psychosocial factors like tooth brushing pattern and oral bad breath met minimum requirement. Substances like current cigarette use, current and life time alcohol use were fulfilled the minimum requirements (p < 0.2 significance level) for further multivariable logistic analysis. In multivariable logistic analysis for depression, the model adequately fits the data as *p* value from Hosmer and Lemeshow test was 0.773. Throughout multivariable logistic analysis, variables like female sex, elementary educational status, periodontitis, history of repeat visit to dental clinic, current alcohol and cigarette use and irregular tooth brushing pattern were significantly associated with depression.

According to this study being female was about 2.22times [AOR 2.22 (95% CI 1.53, 3.23)] more likely to depression as compared with male. From the study participant being elementary educational status were about 2.15times [AOR 2.15 (95% CI 1.28, 3.58)] more likely to develop depression as compare to college/university status participants. With regard to those who have periodontitis were 1.74times [AOR 1.74 (95% CI 1.18, 2.72)] higher to have depression as compared to those who have pulpits. Individuals who have repeated visit to dental clinic were about four-fold higher [AOR 4.07 (95% CI 2.84, 5.84)], as compared to those who have history of newly visiting dental clinic. Individuals who were reported current use of alcohol was 4.01times [AOR 4.01 (95% CI 2.68, 6.00)] more likely to develop depression as compared with non-users. The odds of developing depression among current cigarette smokers were 3.15 times [AOR 3.15 (95% CI 1.42, 7.00] more likely as compared with non-smokers. We had also found that, individuals who have irregular tooth brushing pattern were 2.22times [AOR 2.22 (95% CI 1.53, 3.23] more likely to develop depression as compared with individuals who have regular tooth brushing pattern as shown in Table 4.

**Table 4:** Bi-variable and multivariable logistic analysis of associated factors with depression among people with dental disease in outpatient department at Addis Ababa public Hospitals, Addis Ababa, Ethiopia, 2019 (n = 833)

Variables	Category	Depression		COR(95% CI)	AOR(95%, CI)
		Yes	No		
Sex	Female	157 (18.8%)	309 (37.0%)	1.66 (1.22–2.26)	2.22 (1.53–3.23) **
	Male	86 (10.3%)	281 (33.7%)	1.00	1.00
Educational status	No formal education	32 (3.8%)	89 (10.6%)	1.32 (0.77–2.25)	1.18 (0.62–2.26)
	Elementary	95 (11.4%)	147 (17.6%)	2.38 (1.54–3.67)	2.14 (1.28–3.58) *
	High School	76 (9.1%)	207 (24.8%)	1.35 (0.87–2.09)	1.33 (0.79–2.22)
	College/University	40 (4.8%)	147 (17.6%)	1.00	1.00
Type of Dental disease	Periodontitis	162 (19.4%)	321 (38.5%)	1.57 (1.09–2.29)	1.74 (1.12–2.72) *
	Root stamp	13 (1.5%)	54 (6.4%)	0.75 (0.38–1.49)	0.94 (0.43–2.05)
	Gingivitis	19 (2.2%)	62 (7.4%)	0.96 (0.52–1.76)	0.95 (0.47–1.92)
	Pulpitis	49 (5.8%)	153 (18.6%)	1.00	1.00
Tooth brushing pattern	Irregular	160 (19.2%)	330 (39.6%)	1.83 (1.34–2.49)	2.22 (1.54–3.22) **
	Regular	83 (9.9%)	287 (34.4%)	1.00	1.00
History of visiting dental clinic	Repeat	131 (15.7%)	140 (16.8%)	3.76 (2.74–5.16)	4.01 (2.84–5.83) **
	New	112 (13.4%)	450 (54.0%)	1.00	1.00
Current alcohol	Yes	136 (16.3%)	117 (14.0%)	5.14 (3.71–7.11)	4.00 (2.68–6.00) **
	No	107 (12.8%)	473 (56.7%)	1.00	1.00
Current cigarette	Yes	23 (2.7%)	14 (1.6%)	4.30 (2.17–8.51)	3.15 (1.42–7.00) *
	No	220 (26.4%)	576 (69.1%)	1.00	1.00
Life time alcohol use	Yes	106 (12.7%)	128 (15.3%)	2.79 (2.03–3.85)	1.49 (0.98–2.26)
	No	137 (16.4%)	462 (55.4%)	1.00	1.00
Oral bad breath	Yes	77 (9.2%)	147 (17.6%)	1.39 (1.01–1.94)	1.17 (0.79–1.74)
	No	166 (19.9%)	443 (53.1%)	1.00	1.00
Average monthly income	Below poverty line(< 1627 Ethiopian birr)	123 (14.7%)	267 (32.0%)	1.24 (0.92–1.67)	1.07 (0.74–1.55)
	Above poverty line(≥ 1627 Ethiopian birr)	120 (14.4%)	323 (38.7%)	1.00	1.00

^*^Significant association (*p* value < 0.05), **significant association (*p* value < 0.0001), Hosmer and Lemeshow test = 0.773

## Discussion

### Prevalence and factors associated with anxiety among people with dental disease

The prevalence of anxiety in this study was higher than studies conducted in Turkey 20.8% [26], Finland 10% [16], Pakistan 22.5% [14] and USA Virginia 13.4% [18]. The deviation might be due to the variation in study design, data collection tool and sample size; which was cross sectional study done using Modified dental anxiety scale among 250 participants with self-administered questionnaires method in Turkey [26]. Health survey study done using Composite International Diagnostic Interview among (n=5172)participants in Finland [16]. Observational cross sectional study done using HADS scale among sample size of 80 participants with convenient method selection in Pakistan [14]. A comparative survey study and behavioral risk factor survey with sample size of 76,292in USA Virginia [18].

On other hand this finding was lower than studies conducted in Turkey 36% [15], Pakistan 36.8% [27], Malaysia 36.5% [28]. The difference might be due to the deviation in study design, data collection tool and sample size. A cross sectional study carried out among randomly selected 158 patients using depression, anxiety and stress scale

(DASS) scale and self-administered questionnaires in Turkey [15]. A cross sectional study conducted among randomly selected 72 patients using DASS scale in Pakistan [27]. Case control study conducted among 159 purposively selected participants using DASS scale and self-rated questionnaire in Malaysia [28].

In this study female was more likely to develop anxiety than male. These gender differences in morbidity of anxiety are consistent with many research literatures. Previous research has emphasized that difference in anxiety scores of both males and females is due to the fact that it is easier for females to admit about their anxiety. This difference in anxiety is attributed to female’s lack of power in society, difference in gender role socialization in which it is acceptable for women to report about fears and anxiety [29]. Another study revealed that females are highly responsive in attitude to the anticipated dangers induced by overprotective and over-restrictive parental attitudes or failure to acquire educational, social and vocational abilities or lack of assertiveness or dispositional timidity which acts to magnify their anxiety in relation to inner and outer threats [30]. Previous studies reported that women tend to be more anxious than men [31].

Current finding implied that, environmental setup plays important role in creating female gender more prone to anticipate emotional or psychological stress that lead to anxiety.

This study revealed that individual who had tooth extraction was more than threefold to develop anxiety as compared with those who have tooth scaling. Similarly, study result in USA which show tooth removal was three times more possible to develop anxiety [32]. Studies showed that removal of the tooth causes a decreased capability to feed and potentially influence general health and poor oral hygiene. Mouth and teeth have also social, psychological and cultural significance due to their importance in verbal and nonverbal communication [33, 34]. The current finding might be due to tooth extraction causes stressful experience and painful sensation among the subjects. From another point of view, individuals with tooth extraction might be influenced by local anesthesia, treatment outcome and position of extraction.

The odds of having chronic diseases were more likely to develop anxiety than no having chronic diseases. Chronic diseases such as various forms of oral cancer, diabetes, hypertension and cardiac diseases are at greater risk for people with dental disease which cause more nervous. Studies suggest that coexisting with poor oral health or other chronic conditions with mental disorders contribute series of disease and poorer outcomes among people with dental disease [35]. This might be due to chronic disease play important role in influencing the course and treatment outcome of the subjects which in turn lead to anxiety.

This study revealed that individuals with repeated visit to dental clinic were tripled to develop anxiety as compared with individuals having history of newly visiting dental clinic.

This finding was in line with the study conducted in England revealed factors that might have contributed to anxiety before and, after minor oral surgery/dental procedures and, found that the difficulty of the procedure does not influence the anxiety, but those during follow up period possibly as a result of post-operative complications were develop anxiety [36]. Current study support that, during follow up period there might be post-operative complication such as inflammation, bleeding, pain and swelling. Hence these conditions lead subjects to unpleasant experience and psychological distress that further lead to anxiety. Despite this, study in Spain reported that less anxiety a week after the operation; this may result from rapid recuperation, given that no patients developed any complications [37].

In this study, the odds of alcohol use were more likely to develop anxiety than non-users. Study finding in USA strongly suggest that alcohol abuse can cause depression, anxiety, psychosis and antisocial behavior both during intoxication and withdrawal [38]. Another study showed that acute or chronic alcohol consumption affects multiple neurotransmitter system in the brain that virtually affects brain function [39]. Across time, repeated withdrawal episodes can result in a progressive neural adaptation that makes the drinker more susceptible to anxiety and exacerbates stress-induced negative effect when alcohol intake stops [40]. Hence the result of this study revealed that those conditions may lead to anxiety.

### Prevalence and factors associated with depression among people with dental disease

This study showed that, the prevalence of depression was almost in line with study result in Brazil 28.5% [41]. However, this study was higher than studies in Finland 26% [16], in Pakistan 11.2% [14], in Sweden 4.8% [13] and studies in USA 8% and 16.7%respectively [18, 42]. The discrepancy might be due to variation in study design, data collection tool and sample size. Survey study design and two stage stratified cluster sample of 8028 with structured interview conducted using 21-item modification of beck depression scale (BDI) in Finland [16]. Observational cross sectional study design was done using HADS scale selecting 80 participants in Pakistan [14]. A cross sectional based cohort study was among randomly selected 221 participants using HADS in Sweden [13]. Population based cross sectional and comparative study were conducted using PHQ-9 with cutoff score ≥10 and Behavioral Risk factors survey (BRFS) in USA respectively [18, 42].

Conversely, this study result was lower than study done in Turkey 41% [15], Pakistan 31.64% [27] and Sweden 46% [43]. The difference might be duet to study design, data collection tools and sample size.

Turkey was used clinical based cross sectional study by using Beck depression inventory scale (BDI) with cutoff score ≥10 [15]. Pakistan was used DASS and a cross sectional study design with randomly selected 72 participants [27]. Clinical study conducted among consecutively selected sample of 148 using HADS with 7/8 cut-off score in Sweden [43].

From the study variables view, in adjusted odds ratio most of factors were associated with depression. Along with the variables such as sex, elementary educational status, periodontitis, history of repeat visit to dental clinic, irregular tooth brushing pattern, current alcohol and cigarette use were those significantly associated with depression.

With respect to gender, female was more likely to develop depression as compared to male. This study was supported by studies; women have a greater lifetime risk for the depression as compared to men [30]. From another point of view, from the time girl reaches puberty until age of menopause, female is twice as likely to have depression disorder as compared a male. Depressive disorders also occur earlier in women than in men. This is due to differences in neuron chemical and as a result of the action of estrogen and progesterone [44]. The possible reason might being female is more prone to suppress their emotion or not disclose the status of their problem which in turn debilitate their motives.

The current finding showed that depression was more likely observed among elementary educational status individuals as compared with college/university status. This result finding supported by study done in Finland in which primary educational status was associated with depression [16]. The possible explanation is that, those who educated elementary level might have low coping style for mental illness as compared to college/university level.

In this study, an individual with periodontitis was more likely to develop depression as compared with pulpitis. Study conducted in USA revealed that there is evidence supporting the association mental disorders with physiological and behavioral precursors of periodontal disease which linked with physiological responses associated with mental disorders may reduce salivary flow due to sympathetic stimulation and abnormal immune involved in pathogenesis of periodontal disease, as the alteration of hypothalamic pituitary adrenal axis [45, 46]. Also some behavioral changes associated with psychological distress like depression such as poor oral hygiene which affects periodontal plaque and periodontal disease [47]. This finding also supports previous findings that depression was significantly associated with periodontitis. This might be due to periodontitis may change the course and treatment outcome of the illness.

This study finding revealed that individuals who have history of repeated visit to dental clinic were more likely to develop depression as compared with newly visiting participants.

Study in Finland showed that subjects with a higher number of depressive symptoms had more dental treatment need, but has a lower frequency of dental visit [16]. Another population based study of 55 years old women from Northern Finland, women with depressive symptoms takes a longer time for following at dental clinic when compared with non-depressive women [48]. Study finding in USA, adult with depression was less likely to have used the service of a dental health professional [32]. In this finding depression might be related with negative consequences of dental complication, severity of the disease and medication effect.

The present study showed that an individual with irregular tooth brushing pattern was more than twice to develop depression as compared with regular tooth brushing individuals. The study finding conducted in Turkey revealed that individuals with high depressive scores neglected their oral hygiene, confirming one of the behavioral symptoms of depression (unwillingness to take part in physical activities) [49]. Another study support that an association between the frequency of tooth brushing and depression when the findings were adjusted the participants’ educational level [50]. The other study conducted in Finland found that subjects with a higher number of depressive symptoms had a lower tooth brushing frequency [17]. This study result also supports previous findings. The possible explanation is that poor dental health habits might be mask individuals psychological and physical activities. Hence this might be led to depression.

We found a significant association between depression and alcohol use. The study in USA and Roman has revealed that unhealthy lifestyles, such as alcohol consumption have been shown to be risk factors for depression [51, 52].

Study in New Zealand revealed that the presence of either disorders doubled the risk of the second disorder [53]. This might be due to alcohol use increase the severity of the disease and poor coping style.

Study result in India revealed that cigarette smoking is associated with depression [19]. Also study conducted in USA showed that smoking was associated with a nearly two-fold increased risk of depression relative to both never smokers and former smokers [54]. This might be due to cigarette use play important role in influencing oral hygiene and lead to unhealthy life style.

## Limitation of study

This study was institutional based cross-sectional design. Thus, we cannot infer cause and effect. In fact, the relationship between depression and anxiety, and oral health impact or dental disease may be bidirectional. Lastly, this study finding cannot represent inpatient dental populations.

## Conclusions

In the current study anxiety and depression were high among people with dental disease. Tooth extraction and having chronic disease were significantly associated with anxiety. Elementary educational status, periodontitis, current cigarette smoking and irregular tooth brushing pattern were significantly associated with depression. History of repeat visit to dental clinic, current alcohol use and female sex were significantly associated with both depression and anxiety. So concerned bodies should focus on patients who have chronic disease, those who have tooth extraction, poor oral hygiene (tooth brushing), and who are using substances (alcohol and cigarette) and it is better to screen anxiety and depression at dental clinic. We also recommend researchers inorder to conduct longitudinal study to investigate the cause effect relationship of risk factors of depression and anxiety.

## Data Availability

The datasets used for analysis are available from the corresponding author upon reasonable request.

## Abbreviations

AOR: Adjusted Odds Ratio
BDI: Beck Depression Inventory
BRFS: Behavioral Risk Factor Scale
CI: Confidence Interval
COR: Crude Odds Ratio
DASS: Depression Anxiety Stress Scale
GDS: Geriatrics Depression Scale
HADS: Hospital Anxiety and Depression Scale
NHANES: National Health and Nutrition Examination Survey
PHQ-9: Patient Health Questionnaire-9
SPSS: Statistical Package Software for Social Sciences
USA: United States of America
USD: United States Dollar
WHO: World Health Organization

## References

[1] Vos T, Abajobir AA, Abate KH, Abbafati C, Abbas KM, Abd-Allah F, Abdulkader RS, Abdulle AM, Abebo TA, Abera SF (2017). Global, regional, and national incidence, prevalence, and years lived with disability for 328 diseases and injuries for 195 countries, 1990–2016: a systematic analysis for the Global Burden of Disease Study 2016. Lancet.

[2] Petersen PE, Bourgeois D, Ogawa H, Estupinan-Day S, Ndiaye C (2005). The global burden of oral diseases and risks to oral health. Bull World Health Organ.

[3] Organization WH: International statistical classification of diseases and related health problems, vol. 1: World Health Organization; 2004. 10.2471/BLT.25.294650 PMC13531092 42683092

[4] Association AP: Diagnostic and statistical manual of mental disorders (DSM-5®): American Psychiatric Pub; 2013. 10.1590/s2317-17822013000200017 24413388

[5] Sadock BJSaVA: Synopsis of Psychiatry, Behavioral Sciences/Clinical Psychiatry; 2015.

[6] Organization WH (2017). Depression and other common mental disorders: global health estimates.

[7] Strine TW, Mokdad AH, Balluz LS, Berry JT, Gonzalez O (2008). Impact of depression and anxiety on quality of life, health behaviors, and asthma control among adults in the United States with asthma, 2006. J Asthma.

[8] Olatunji BO, Cisler JM, Tolin DF (2007). Quality of life in the anxiety disorders: a meta-analytic review. Clin Psychol Rev.

[9] Marya C, Grover S, Jnaneshwar A, Pruthi N (2012). Dental anxiety among patients visiting a dental institute in Faridabad, India. West Indian Med J.

[10] D'Mello D (2003). Are your patients depressed? Implications for dental practice. J Mich Dent Assoc.

[11] Feinmann C, Harris M, Cawley R (1984). Psychogenic facial pain: presentation and treatment. Br Med J (Clin Res Ed).

[12] Miyachi H, Wake H, Tamaki K, Mitsuhashi A, Ikeda T, Inoue K, Tanaka S, Tanaka K, Miyaoka H (2007). Detecting mental disorders in dental patients with occlusion-related problems. Psychiatry Clin Neurosci.

[13] Stenebrand A, Wide Boman U, Hakeberg M (2013). Dental anxiety and symptoms of general anxiety and depression in 15-year-olds. Int J Dental Hygiene.

[14] Khan MA (2015). Anxiety and Depression in Patients Attending Institute of Dentistry CMH Lahore. Ann Pakistani Inst Med Sci.

[15] Dirik G, Kilicarslan MA, Gençöz T, Karanci N (2006). Correlates of anxiety and depression in Turkish complete denture patients. Soc Behav Personal Int J.

[16] Delgado-Angulo EK, Sabbah W, Suominen AL, Vehkalahti MM, Knuuttila M, Partonen T, Nordblad A, Sheiham A, Watt RG, Tsakos G (2015). The association of depression and anxiety with dental caries and periodontal disease among Finnish adults. Commun Dent Oral Epidemiol.

[17] Anttila S, Knuuttila M, Ylöstalo P, Joukamaa M (2006). Symptoms of depression and anxiety in relation to dental health behavior and self-perceived dental treatment need. Eur J Oral Sci.

[18] Wiener RC, Wiener MA, McNeil DW (2015). Comorbid depression/anxiety and teeth removed: behavioral risk factor surveillance system 2010. Commun Dent Oral Epidemiol.

[19] Shrestha S, Sharma S, Sapkota N, Giri DK, Baral D (2017). Association between anxiety and depression with chronic periodontitis. J Coll Med Sci Nepal.

[20] Dumitrescu AL (2016). Depression and inflammatory periodontal disease considerations—an interdisciplinary approach. Front Psychol.

[21] Hugo F, Hilgert J, De Sousa M, Cury J (2012). Depressive symptoms and untreated dental caries in older independently living South Brazilians. Caries Res.

[22] Accorsi S, Bilal NK, Farese P, Racalbuto V (2010). Countdown to 2015: comparing progress towards the achievement of the health Millennium Development Goals in Ethiopia and other sub-Saharan African countries. Trans R Soc Trop Med Hyg.

[23] Reda AA (2011). Reliability and validity of the Ethiopian version of the hospital anxiety and depression scale (HADS) in HIV infected patients. PLoS ONE.

[24] Abiola T, Udofia O, Zakari M (2013). Psychometric properties of the 3-item oslo social support scale among clinical students of Bayero University Kano, Nigeria. Malays J Psychiatry.

[25] Method WBa: Country and lending groups: Low income economies Low income economies 2017.

[26] Pekkan G, Kilicoglu A, Hatipoglu H (2011). Relationship between dental anxiety, general anxiety level and depression in patients attending a university hospital dental clinic in Turkey. Community Dent Health.

[27] Rashid H, Hussain SS. Prevalence of depression, anxiety and stress among orthodontics patients visiting a tertiary care hospital, pakistan. Int J Dental Clin. 2014;6(1).

[28] Radeef A, Faisal G (2017). Assessment of depression, anxiety and stress symptoms among patients with periodontal disease. J Int Dental Med Res.

[29] Arrindell WA, Eisemann M, Richter J, Oei TP, Caballo VE, Van der Ende J, Sanavio E, Bagés N, Feldman L, Torres B (2003). Masculinity–femininity as a national characteristic and its relationship with national agoraphobic fear levels: Fodor’s sex role hypothesis revitalized. Behav Res Ther.

[30] Farooqi YN, Ahsan S (2009). Gender differences in anxiety and depression among Pakistani cancer patients. J Res Soc Pak.

[31] Yildirim TT, Dundar S, Bozoglan A, Karaman T, Dildes N, Kaya FA, Altintas E, Oztekin F, Atas O, Alan H (2017). Is there a relation between dental anxiety, fear and general psychological status?. PeerJ.

[32] Okoro CA, Strine TW, Eke PI, Dhingra SS, Balluz LS (2012). The association between depression and anxiety and use of oral health services and tooth loss. Commun Dent Oral Epidemiol.

[33] United States. Public Health Service. Office of the Surgeon General, National Institute of Dental, Craniofacial Research (US). Oral health in America: a report of the Surgeon General. US Public Health Service, Department of Health and Human Services;2000.

[34] Joshipura K, Ritchie C, Douglass C: Strength of evidence linking oral conditions and systemic disease. Compendium Contin Educ Dent (Jamesburg, NJ: 1995) Supplement 2000(30):12–23; quiz 65. 11908384

[35] Satcher D (2000). Mental health: a report of the Surgeon General-Executive summary. Prof Psychol Res Pract.

[36] Earl P (1994). Patients' anxieties with third molar surgery. Br J Oral Maxillofac Surg.

[37] López-Jornet PC-AF, Sanchez-Siles M (2014). Assessment of general pre and postoperative anxiety in patients undergoing tooth extraction: a prospective study. Br J Oral Maxillofac Surg.

[38] Shivani R, Goldsmith RJ, Anthenelli RM (2002). Alcoholism and psychiatric disorders: diagnostic challenges. Alcohol Res Health.

[39] Schulte T, Oberlin BG, Kareken DA, Marinkovic K, Müller-Oehring EM, Meyerhoff DJ, Tapert S (2012). How acute and chronic alcohol consumption affects brain networks: insights from multimodal neuroimaging. Alcoholism Clin Exp Res.

[40] Breese GR, Overstreet DH, Knapp DJ (2005). Conceptual framework for the etiology of alcoholism: a “kindling”/stress hypothesis. Psychopharmacology.

[41] Ávila GÁdC, Martins AB, D'AVILA OP, Neves M, Hilgert JB, Hugo FN: Association between depressive symptoms and dental care-seeking behavior among elderly Brazilian people. *Revista de Odontologia da UNESP* 2016, 45(3):132–138.

[42] O’Neil A, Berk M, Venugopal K, Kim S-W, Williams LJ, Jacka FN (2014). The association between poor dental health and depression: findings from a large-scale, population-based study (the NHANES study). Gen Hosp Psychiatry.

[43] Bohman W (2010). Psychosocial and dental factors in the maintenance of severe dental fear. Swed Dent J.

[44] Dalgard OS, Dowrick C, Lehtinen V, Vazquez-Barquero JL, Casey P, Wilkinson G, Ayuso-Mateos JL, Page H, Dunn G (2006). group O: Negative life events, social support and gender difference in depression. Soc Psychiatry Psychiatr Epidemiol.

[45] Elter JR, Beck JD, Slade GD, Offenbacher S (1999). Etiologic models for incident periodontal attachment loss in older adults. J Clin Periodontol.

[46] Kasper S, Den Boer JA, Sitsen JA (2003). Handbook of depression and anxiety: a biological approach.

[47] Pihlstrom BL, Michalowicz BS, Johnson NW (2005). Periodontal diseases. Lancet.

[48] Sisko Anttila S, Knuuttila MLE, Sakki TK (2001). Relationship of depressive symptoms to edentulousness, dental health, and dental health behavior. Acta Odontol Scand.

[49] Alkan A, Cakmak O, Yilmaz S, Cebi T, Gurgan C (2015). Relationship between psychological factors and oral health status and behaviours. Oral Health Prev Dent.

[50] Marques-Vidal P, Milagre V (2006). Are oral health status and care associated with anxiety and depression? A study of Portuguese health science students. J Public Health Dent.

[51] Paperwalla KN, Levin TT, Weiner J, Saravay SM. Smoking and depression. Med Clin N Am 2004, 88(6):1483–1494, x-xi. 10.1016/j.mcna.2004.06.007 15464109

[52] Kieres-Salomoński I, Wojnar M (2015). Comorbidity of alcohol dependence with other psychiatric disorders. Part I. Epidemiology of dual diagnosis. Psychiatr Pol.

[53] Boden JM, Fergusson DM (2011). Alcohol and depression. Addiction.

[54] Luger TM, Suls J, Vander Weg MW (2014). How robust is the association between smoking and depression in adults? A meta-analysis using linear mixed-effects models. Addict Behav.

